# Preparation of Layered Polyethylene Oxide/rGO Composite: Flexible Lateral Heat Spreaders

**DOI:** 10.3390/polym11030532

**Published:** 2019-03-21

**Authors:** Fubin Luo, Pinping Yan, Qingrong Qian, Hongzhou Li, Baoquan Huang, Qinghua Chen

**Affiliations:** 1 College of Environmental Science and Engineering, Fujian Normal University Fuzhou 350007, China; luofubin@fjnu.edu.cn (F.L.); pinpingyan@126.com (P.Y.); qbh811@sina.com (B.H.); cqhuar@126.com (Q.C.); 2 Engineering Research Center of polymer Green Recycling of Ministry of Education, Fuzhou 350007, China; 3 Fujian Key Laboratory of Pollution Control & Resource Reuse, Fuzhou 350007, China

**Keywords:** thermal conductivity, graphene, polyethylene oxide

## Abstract

In this paper, high thermal conductive polyethylene oxide (PEO)/reduced graphene oxide (rGO) composite is prepared via large-scale green reduction. Flexible layered PEO/GO composites are pre-prepared in aqueous solution. It is demonstrated that PEO chains can form hydrogen bonds with GO. Being driven by hydrogen bonds, GO/PEO composites show homogeneous and lateral highly oriented structures, resulting in excellent mechanical properties. The pre-prepared composite films are large scale soaked into ascorbic acid solution. GO nanosheets in the matrix of the composites can be reduced by ascorbic acid. The results indicate that PEO chains can repair the damage of the films caused by the reduction process. Therefore, the films can maintain their original configuration and still keep excellent flexibility. By comparison, pristine GO films are totally destroyed when the same reduction is experienced. Due to the presence of PEO, the lateral highly oriented structure of the composite will not be damaged. After reduction, the thermal conductivity of the composite reaches to 12.03 W m^−1^ K^−1^ along the rGO nanosheet oriented direction.

## 1. Introduction

High-power electronic devices can generate large amounts of heat when they run. Heat can be rapidly accumulated in a narrow space if it is not removed, which can shorten the service lifetimes and influence the signal transmission of the devices [1,2,3]. Therefore, it is urgent to develop heat-dissipating materials for highly integrated circuits. Polymers, such as epoxy, silicone rubber, and polyimide, are usually used for the preparation of high thermal conductive materials [4,5,6,7,8]. However, it is hard to satisfy the requirement of heat dissipation with polymers, because most polymeric materials possess an extremely low thermal conductive coefficient (≤0.5 W m^−1^ K^−1^). To achieve high thermal dissipating ability, polymers filled with inorganic fillers are a regular solution [9,10,11,12]. The issue is that the advantage of polymers would be destroyed upon the addition of fillers with high volume contents. It seems that achieving high thermal diffusion conflicts with keeping moldability and excellent mechanical properties of polymer composites [13].

Graphene is an attractive two-dimensional (2D) carbon material with carbon atoms organized in a hexagonal lattice structure. Graphene is a promising candidate for improving the thermal conductivity of polymers because of its intrinsic ultrahigh thermal conductivity [14,15,16,17]. In spite of this, it has proven to be difficult to realize solution-based handling and large-scale direct synthesis [18,19]. A functionalized form of graphene, graphene oxide (GO), can be converted into graphene via chemical reduction, called reduced graphene oxide (rGO) [20]. By comparison, GO can be dispersed in water under neutral or basic conditions, and it is more facile to be directly synthesized on a large scale [21,22,23,24]. While the thermal conductivity of GO is far from satisfactory, according to the previous reports, the thermal conductivity of GOs is less than 1 W m^−1^ K^−1^ [25]. Some studies report the thermal conductivity of GO paper [26,27]. The chemical reduction of GO is an effective route to obtain relatively high thermal conductive graphene. The common reduction agent is hydrazine, hydroiodic acid, which is considered to be toxic, corrosive, and hazardous. Recently, researchers have developed a “green reduction” approach to synthesize rGO, resembling pristine graphene to a larger extent. It has been proven that antioxidants, organic acids, plant extracts, amino acids, etc. can reduce GO [28,29,30].

Attempts to prepare heat-dissipating materials based on rGO have achieved some progress [31,32,33]. Annealing GO at an ultra-high temperature was confirmed to be an effective route [34,35]. Moreover, rGO is usually filled into another matrix for the purpose of high heat dissipation [36,37,38,39]. In this work, aiming to prepare high thermal conductive material, ascorbic acid is used as a reduction agent for GO. The fabrication process of flexible, high thermal conductive film is environmentally friendly in an aqueous solution system. Supported with low-content water-soluble polyethylene oxide (PEO), the prepared film can keep its integrity, as well as its excellent flexibility and mechanical properties. The fabrication mechanism and the thermal diffusion of the composite are investigated.

## 2. Experiments

### 2.1. Materials

Graphite powder (99.95%) and ascorbic acid were purchased from Aladdin (Shanghai, China). Polyethylene oxide (PEO, *Mw* = 600,000) was provided by Guangzhou Liguo Trading Co., Ltd. (Guangzhou, China).

### 2.2. Synthesis of Graphene Oxide (GO)

GO was prepared by a chemical oxidation route based on the modified Hummers’ method [40,41]. As per our previous report [42], the prepared GO was processed by centrifugation and dialysis.

### 2.3. Synthesis of the Composites

GO/PEO films: GO solution was prepared and sonicated for 30 min. Next, PEO was added, followed by 3 h of magnetic stirring. The mixture was poured into the prepared flat Teflon mold, and then, the water was evaporated at room temperature until the film was able to be peeled off the mold. Finally, the film was dried at 60 °C. According to the stoichiometric ratios of GO and PEO, the samples were marked as GO/PEO-1, GO/PEO-2, and GO/PEO-3, which showed that the mass ratio of GO to PEO is 20:1, 20:4, and 20:10.

Reduction: Ascorbic acid solution (10 mg/mL) was prepared. GO/PEO films were immersed into the solution for 80 h. In order to guarantee the reduction agent was sufficient, about 0.1 g of composite films were immersed into 500 mL of solution. After that, the films were collected and dried at 60 °C for about 10 h. The reduced films were named GO/PEO-R1, GO/PEO-R2, and GO/PEO-R3.

### 2.4. Material Characterization

The morphological characterization was done by a Hitachi S3400N (Tokyo, Japan) scanning electron microscope (SEM). The energy dispersive spectroscopy was measured using SEM equipped with an energy dispersive spectroscopy (EDS) detector. To monitor structural changes, such as the degree of defects, Raman spectroscopy was performed by Raman spectrometer (Renishaw, London, UK) excited at 632.8 nm. The XRD patterns were collected using a Rigaku D/MAX-1200 X-ray diffractometer with Cu Ka radiation (k = 0.154 nm) at room temperature in the range of 1.5–80°. The mechanical properties were tested on a computer-type tensile testing machine, and the stress-strain curves were recorded. The thermal conductivity was calculated by the following formula: *K* = *ρC_p_*λ, where *K* is the thermal conductivity value of the sample. *ρ*, *C_p_*, and λ represent the density, specific heat capacity, and thermal diffusion coefficient, respectively. The measurements of cross-plane and in-plane thermal diffusion λ were performed using an LFA laser flash apparatus (Netzsch LFA 477, Selb, Germany). Two types of test specimens were used, and both had a thickness of about 40 μm; the “in-plane” circular samples had a diameter of 25.4 mm, and the “through-plane” circular samples had a diameter of 12.7 mm. The *C_p_* was measured by differential scanning calorimetry (DSC, NETZSCH DSC 214, Selb, Germany). *ρ* was measured by a density apparatus first and affirmed by the calculation (via mass/volume) of different circular samples.

## 3. Results and Discussion

### 3.1. Morphology Characterization of the Films

**Figure 1:**
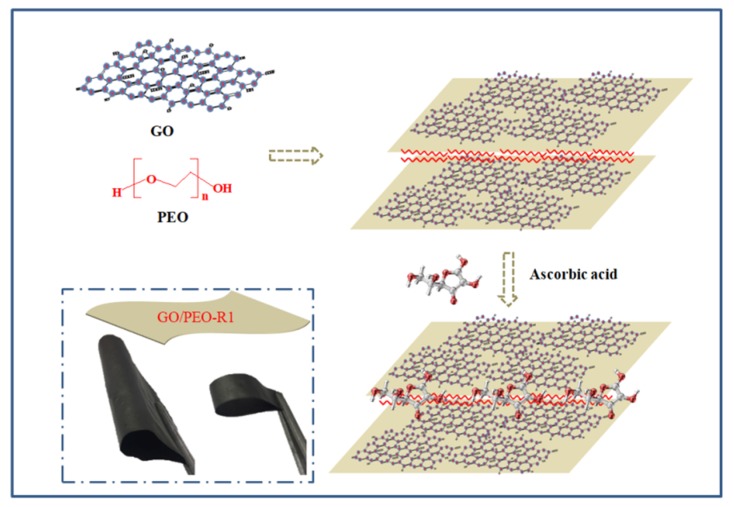
Schematic diagram of reduced graphene oxide film.

**Figure 2:**
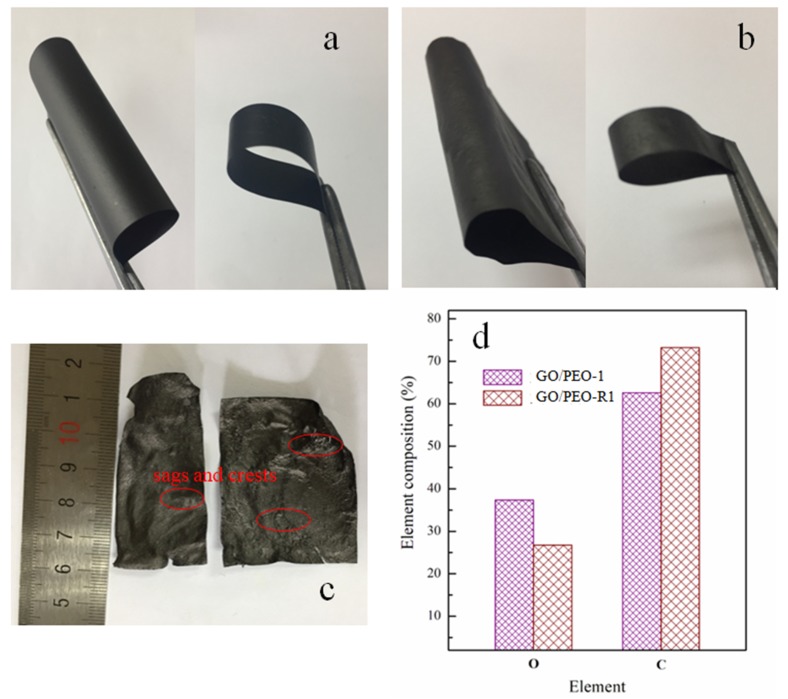
Digital images of (**a**) GO/PEO-1, (**b**) GO/PEO-R1, and (**c**) pristine GO after 80 h reduction in ascorbic acid solution, (**d**) is the element composition of GO/PEO-1 and GO/PEO-R1 evaluated by energy dispersive spectroscopy (EDS).

**Figure 3:**
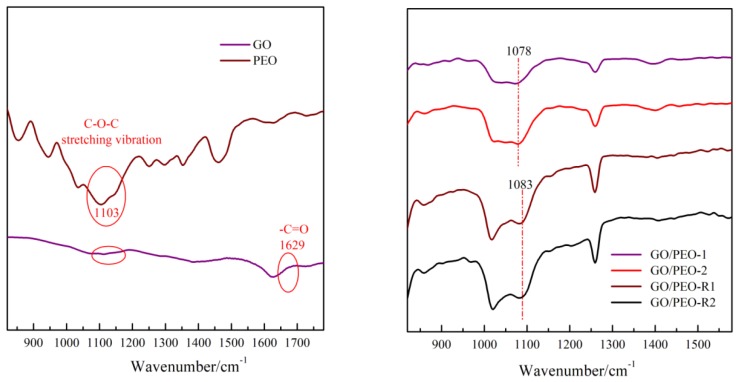
(**a**) FTIR spectra of pristine GO and PEO and (**b**) FTIR spectra of GO/PEO-1 and GO/PEO-2 with and without reduction.

The composition and the corresponding designation of the films are listed in Table 1. Figure 1 depicts the structure diagram and preparation process of the films. The GO/PEO films were pre-prepared by a solution casting method. Figure 2a shows the digital images of the prepared film GO/PEO-1, which is flexible and can bear large amplitude bending. The microstructure of the GO/PEO series film is a nacre-like typical hierarchical layered structure based on SEM investigation. PEO can form a hydrogen bond with GO, which might contribute to the formation of film. The hydrogen bonds in GO/PEO were confirmed by the FTIR spectra. As Figure 3a,b shows, pristine GO reveals weak oxygen-containing functional group absorption spectra. The characteristic peak of PEO located at 1103 cm^−1^ corresponds to C–O–C stretching vibration. The FTIR spectra of GO/PEO-1 and GO/PEO-2 display that peaks of C–O–C stretching vibration shifted to 1078 cm^−1^. It is believed that hydrogen bonds are formed between the GO and PEO molecular chain. After reduction, according to the FTIR spectra, it can be observed that the hydrogen bonds have not been destroyed.

The prepared GO/PEO films were immersed in ascorbic acid solution for 80 h. After undergoing reduction, the films still kept their integrity, as well as their excellent flexibility and mechanical properties. The mechanical flexibility is displayed in Figure 2b. The ascorbic acid molecule is expected to permeate into the composites, accompanied by the reduction reaction. The element compositions of GO/PEO-1 and GO/PEO-R1 demonstrate that the reduction process leads to the decrement of oxygen content, which might be attributed to the removal of oxygen-containing groups. Pre-preparation GO/PEO film followed by reduction is a method that can take full advantage of the excellent compatibility between GO and PEO. It is difficult to obtain high-performance composite film with the direct blending of rGO and PEO. The reason for this might be that the removal of functional groups has an adverse effect on the dispersion of GO in an aqueous solution, as well as on its compatibility with PEO.

**Figure 4:**
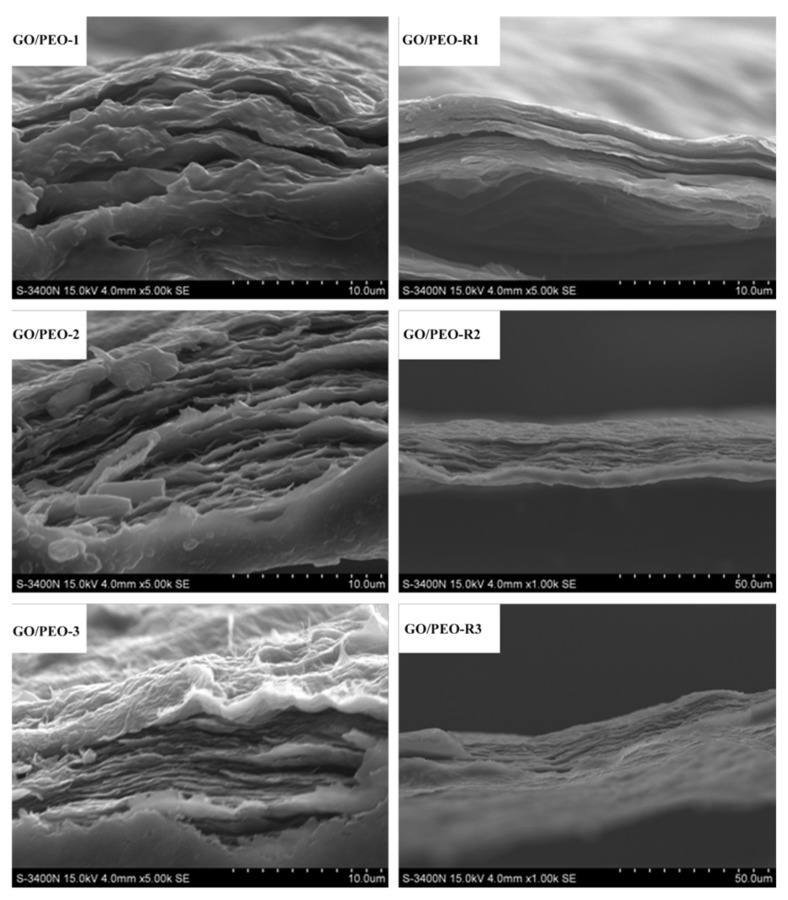
SEM images of the films from section view.

It is noted that pristine GO, after undergoing reduction, becomes rough and brittle. As Figure 2c shows, surfaces with corrugations can be observed. The fabrication of GO film mainly relies on the orientated stacking of every GO nanosheet, which is connected by hydrogen bonding between the oxygen-containing functional group. The reduction reaction might eliminate the functional group and destroy the chemical bonding. As a result, reduced GO film loses its flexibility and mechanical properties. The reduction process of GO film might take place in the same way as that of the composite film. The difference, however, might be that the soluble PEO might immediately repair the deficit or gaps left by reduction. Because PEO is water soluble, the molecular chains can wriggle, resulting in PEO being trapped by the deficits or gaps in the interlayer space of the composite film. As Figure 4 shows, based on the SEM observation from the section view, GO/PEO-R1 also has a laminate structure, in which the reduced GO nanosheets present a long-range, well-packed, layered configuration. This supports PEO, GO/PEO-R1, GO/PEO-R2, and GO/PEO-R3 to hold excellent flexibility and mechanical properties after reduction (As shown in Figure S1).

### 3.2. Raman and XRD Characterization of the Films

**Figure 5:**
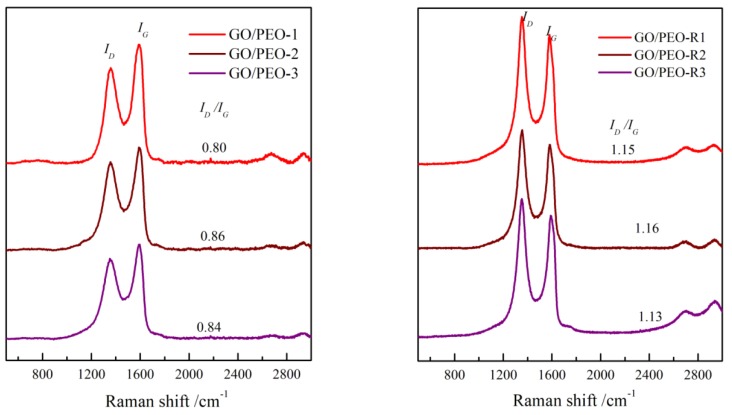
Raman spectra of the films.

Raman spectroscopy is an effective tool employed to characterize carbon-based materials. Figure 5 shows the Raman spectra for GO/PEO-1, GO/PEO-2, GO/PEO-3, GO/PEO-R1, GO/PEO-R2, and GO/PEO-R3. The peaks at 1367 and 1593 cm^−1^ correspond to the D and G peaks. The D peak and G peak of rGO are associated with the disorder in sp^2^-hybridyzed carbons and the first-order scattering of E2g phonon of sp^2^ carbons, respectively [43]. Usually, I_D_/I_G_ (the intensity ratio of D to G) is considered to be an index of the disorder or restoration of the graphene lattice [44]. I_D_/I_G_ of GO/PEO-1, GO/PEO-2, and GO/PEO-3 was 0.80, 0.86, and 0.84, respectively, after reduction, and it is increased to 1.15, 1.16, and 1.13 for GO/PEO-R1, GO/PEO-R2, and GO/PEO-R3, respectively. Theoretically, the reduction of GO can improve the disorder structure, and the graphene-like sp^2^ domain would be increased, resulting the decrement of I_D_/I_G_. Obviously, this fact is opposite to the theoretical prediction. The reason is that the size of the graphene-like sp^2^ domain formed upon reduction is smaller than that of GO, and, as a result, the average size of the sp^2^ domain for reduced GO is decreased, which leads to the decrement of I_D_/I_G_ reflected in the Raman spectra.

**Figure 6:**
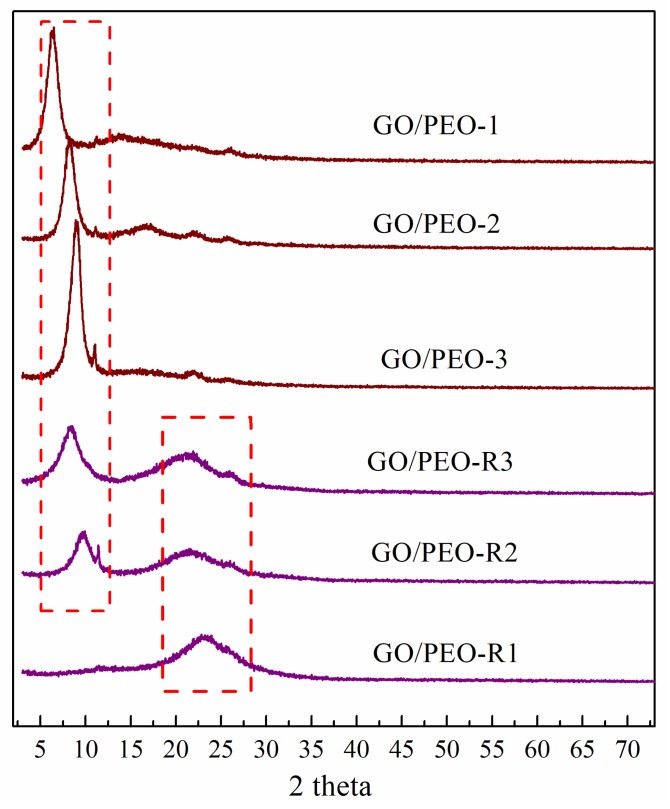
XRD patterns of the films.

In order to further investigate the reduction of the composite films by ascorbic acid, the XRD patterns of the films before and after reduction are displayed in Figure 6. It can be found that GO/PEO-1, GO/PEO-2, and GO/PEO-3 composite films had a sharp diffraction peak at 2θ = around 9°, which is the typical diffraction peak of GO corresponding to the *d* spacing. By comparison, the typical peaks of GO in the XRD patterns of GO/PEO-R1, GO/PEO-R2, and GO/PEO-R3 totally disappeared or shifted. For GO/PEO-R1, the peaks located at around 9° disappear, and instead, a broad peak centered at 2θ = 23.1° was detected, indicating the total reduction of GO in the GO/PEO-1 composite. However, for GO/PEO-2 and GO/PEO-3, except for the broad peak at 2θ = 23.1°, another peak at 2θ =10.1° also arose. This phenomenon demonstrates that GO nanosheets have not been totally reduced by ascorbic acid in GO/PEO-2 and GO/PEO-3 composites.

### 3.3. Thermal Conductivity

The thermal conductivity coefficient (*T_c_*) of the composite film was calculated. According to the result, the *T_c_* of pristine GO was 5.38 and 0.21 in the in-plane direction and out-of-plane direction, respectively, which is consistent with previous reports [26,27]. It can be noted that PEO can increase the *T_c_* of GO paper (As shown in Figure S2). In both directions, GO/PEO-1 and GO/PEO-2 possessed higher *T_c_* compared with GO. This phenomenon is unusual, because a low thermal conductive polymer often has a negative effect on the improvement of the *Tc* in polymer composites. The possible reason is that the PEO chain can form hydrogen bonds with GO. It is believed that hydrogen bonds can significantly improve the thermal conductivity of polymer blends [45,46]. The FTIR results of the films demonstrate the presence of hydrogen bonds in the composites. It is noted that the *T_c_* of GO/PEO-3 is lower than that of GO, indicating that excessive PEO is averse to the improvement of thermal conductivity. In spite of this, hydrogen bonds might exist between the interface of the PEO layer and GO. The thick PEO layer might act as the thermal transport barrier, which results in the lower *T_c_* of the GO/PEO-3 composite film.

**Figure 7:**
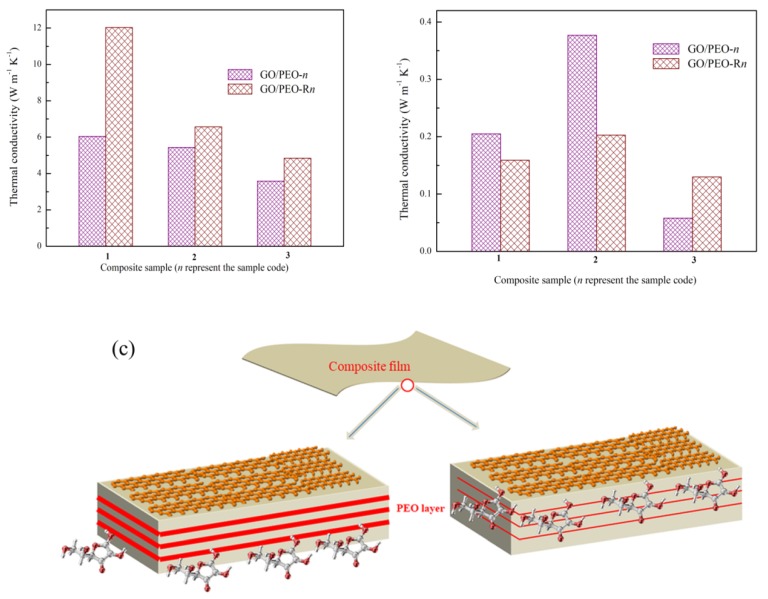
In-plane (**a**) and out-of-plane (**b**) thermal conductivity of GO/PEO composite films with and without reduction; (**c**) reduction diagrams of the GO/PEO composite films.

Figure 7a,b displays the thermal conductivity of the GO/PEO composite films. The reduced films still obtain anisotropic *T_c_*. The SEM observations from the section view of GO/PEO-R1, GO/PEO-R2, and GO/PEO-R3 show that the reduction did not damage the oriented arrangements of the GO nanosheets. The GO/PEO-R1 film exhibits much higher *T_c_* than that of GO/PEO-1 in the in-plane direction, which was increased by 199%. The enhancement originates from repairing the defects of GO by reduction. However, for GO/PEO-R2 and GO/PEO-R3, the *T_c_* only increased by 121% and 135% compared with the corresponding film. The reason might be that the GO nanosheets have not been totally reduced by ascorbic acid in the GO/PEO-R2 and GO/PEO-R3 films, which is consistent with the XRD analysis. The reduction process occurs in an aqueous solution, and the PEO chain can absorb H_2_O molecules during the reduction. Accompanied by the absorption of H_2_O molecules, ascorbic acid molecules are expected to be conveyed into the composites. Therefore, the reduction reaction occurs on the interface of the GO nanosheets. However, when the PEO layer becomes thick (Figure 7c), ascorbic acid molecules might find it difficult to transfer through the layer, leading to the insufficient contact of ascorbic acid molecules and GO. As a result, the reduction reaction cannot be totally completed.

### 3.4. Mechanical Properties

**Figure 8:**
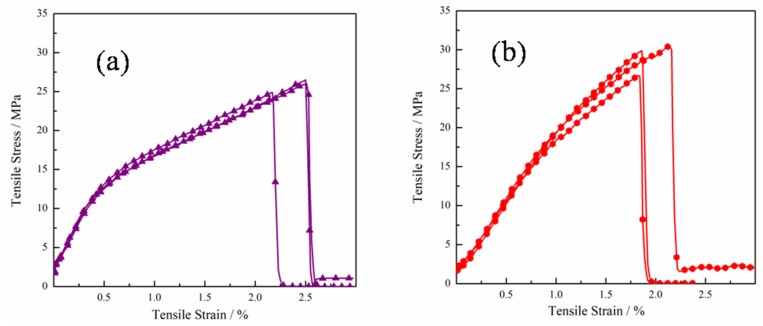
Stress–strain curves of (**a**) GO/PEO-1 and (**b**) GO/PEO-R1.

Figure 8 shows the stress–strain curves of GO/PEO-1 and GO/PEO-R1 composite films. The related data are displayed in Table 2. Both the GO/PEO and GO/PEO-R1 films have excellent mechanical properties. The tensile strength of the GO/PEO-1 and GO/PEO-R1 composite films is 25.8 and 28.7 MPa, respectively, which indicates that the strength has not been impaired by reduction. The superior strength of the GO/PEO-R1 composite films might be derived from the compact laminated structure. Simultaneously, the PEO chain might contribute to the enhancement of tensile strength. On the contrary, the modulus of the GO/PEO-R1 films are much lower than that of the GO/PEO-1 films, which might be decided by the hydrogen bond. After reduction, the hydrogen bond in GO/PEO-R1 is weakened. In addition, the GO/PEO-1 composite films have a relatively high elongation at a break of 2.37. It is apparent that the fracture behavior of GO/PEO-1 and GO/PEO-R1 is different. Based on the tensile–stress curves, it can be found that the GO/PEO-1 composite films undergo a “rubber-like” elongation platform before fracture, which might be attributed to the slippage of the GO sheets.

## 4. Conclusions

In this paper, a series of flexible GO/PEO composites was pre-prepared based on GO. It is demonstrated that PEO chains can form hydrogen bonds with GO. Driven by hydrogen bonding, graphene oxide (GO) and polyethylene oxide (PEO) formed a homogeneous and lateral highly oriented composite in an eco-friendly aqueous solution system. The films are flexible and possess excellent mechanical properties. The tensile strength and modulus are 25.8 MPa and 2.43 GPa, respectively. According to the results, the thermal diffusion of the composite films increased by 112% when containing 5 wt% PEO, which can be attributed to the hydrogen bonds formed between GO and PEO chains. In order to prepare high thermal conductive films, the films were reduced by ascorbic acid. After reduction, the films still kept their integrity, as well as their excellent flexibility and mechanical properties. This could be because the soluble PEO chain might immediately repair the deficits or gaps left by reduction. SEM observations from the section view demonstrate that the reduction did not damage the oriented arrangements of the GO nanosheets. As a result, the reduced films still obtained the anisotropic thermal diffusion coefficient. Due to the green reduction, the thermal conductivity in the in-plane direction was enhanced by199% compared with the GO film, reaching to 12.03 W m^−1^ K^−1^. However, when the content of PEO was increased, the reduction might have been restrained, leading to a relatively small increment of thermal conductivity.

## Figures and Tables

**Table 1:** The composition of the composites. GO graphene oxide; PEO: high thermal conductive polyethylene oxide.

Sample Code	Mass Ratio GO/PEO	Reduction
GO/PEO-1	20:1	without
GO/PEO-2	20:4	without
GO/PEO-3	20:10	without
GO/PEO-R1	20:1	with
GO/PEO-R2	20:4	with
GO/PEO-R3	20:10	with

**Table 2:** The tensile strength of the GO/PEO hybrid film.

Sample	Average Tensile Strength (MPa)	Average Elongation at Break (%)	Average Modulus (GPa)
GO/PEO-1	25.8	2.37	2.43
GO/PEO-R1	28.7	1.90	1.46

## Supplementary Materials

The following are available online at https://www.mdpi.com/2073-4360/11/3/532/s1, Figure S1: Digital image of GO/PEO-R2 and GO/PEO-R3, Figure S2: Thermal conductivity of GO and GO/PEO composite films (a) In-plane, (b) Out of-plane.
