# Supplementary material for: Preparation of Layered Polyethylene Oxide/rGO Composite: Flexible Lateral Heat Spreaders
Source: Polymers (Basel). 2019 Mar 21;11(3):532. doi: 10.3390/polym11030532 (PMC6473242; doi:10.3390/polym11030532)
Supplement: Supplementary file 1 [file polymers-11-00532-s001.pdf]

Article

Preparation of Layered Polyethylene Oxide/rGO Composite: Flexible Lateral Heat Spreaders

Fubin Luo [1,2,3], Pinping Yan [1,2,3], Qingrong Qian [1,2,3,*], Hongzhou Li [1,2,3,*], Baoquan Huang [1,2,3] and Qinghua Chen [1,2,3]

[1] College of Environmental Science and Engineering, Fujian Normal University Fuzhou 350007, China
[2] Engineering Research Center of polymer Green Recycling of Ministry of Education, Fuzhou 350007, China
[3] Fujian Key Laboratory of Pollution Control & Resource Reuse Fuzhou 350007, China
[*] Correspondence: qrqian@fjnu.edu.cn (Q.Q.); lihongzhou@fjnu.edu.cn (H.L.)

1. Digital image of GO/PEO-R2 and GO/PEO-R3

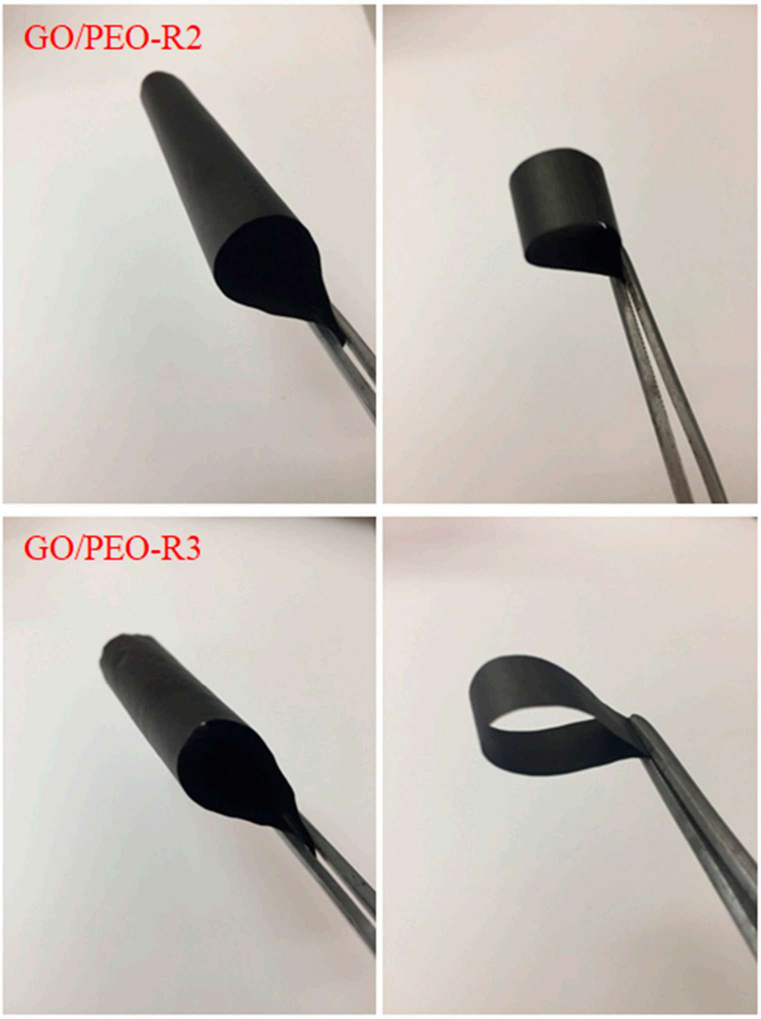

Figure S1. digital image of GO/PEO-R2 and GO/PEO-R3

2. Thermal conductivity of GO and GO/PEO composite films

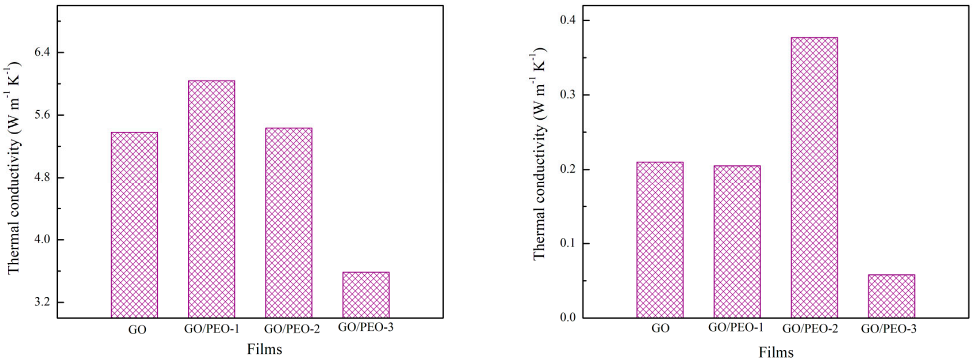

Figure S2. Thermal conductivity of GO and GO/PEO composite films (a) In-plane, (b) Out of-plane
